# P-271. Contact Patterns of United States Health Care Workers at Quaternary Health Center: Stability of contacts during the post-pandemic era

**DOI:** 10.1093/ofid/ofae631.475

**Published:** 2025-01-29

**Authors:** Lauren Pischel, Obianuju G Aguolu, Noureen Ahmed, Melissa Campbell, Ryan C Borg, Chelsea Duckwall, Kathryn Willebrand, Elliott E Paintsil, M Catherine Muenker, Amyn Malik, Moses C Kiti, Samuel Jenness, Ben Lopman, Justin Belsky, Richard A Martinello, Inci Yildirim, Albert I Ko, Saad Omer

**Affiliations:** Yale School of Medicine, New Haven, Connecticut; Ohio State University College of Public Health, Streetsboro, Ohio; Peter O’Donnell Jr. School of Public Health at the University of Texas Southwestern Medical Center, Dallas, Texas; Duke University Medical Center, Durham, North Carolina; Yale University School of Public Health, New Haven, Connecticut; Yale University, New Haven, Connecticut; Yale School of Public Health, Olympia, Washington; Medical College of Wisconsin, Wauwatosa, Wisconsin; Yale School of Public Health, Olympia, Washington; UT Southwestern Medical Center, Dallas, Texas; Emory University, Atlanta, Georgia; Emory University, Atlanta, Georgia; Rollins School of Public Health, Emory University, Atlanta, GA; Yale School of Medicine, New Haven, Connecticut; Yale School of Medicine, New Haven, Connecticut; Yale University, New Haven, Connecticut; Yale School of Public Health/Oswaldo Cruz Foundation/Brazilian Ministry of Health, New Haven, Connecticut; University of Texas South Western, Dallas, Texas

## Abstract

**Background:**

We lack understanding of how health care workers’ (HCWs) contacts change over time or in response to pandemics. This study describes the social contact patterns of HCWs in a large United States health care system via standardized social contact diaries.

Mean number of total contacts over time (Jan 2021-May 2022):

**Figure:**
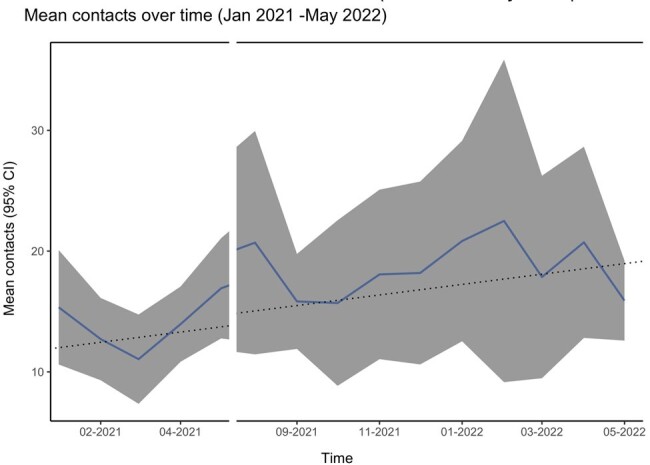

The mean contacts noted with dark-blue line with 95% confidence intervals (95%CI) in dark gray. The dashed black line shows the linear regression of contacts over time. The-axis is interrupted as the study was closed in June and July 2021.

**Methods:**

Inpatient and outpatient HCWs enrolled from October 2020 to June 2022. Participants completed a monthly survey of all contacts during a single representative working day selected by the participant. Direct contact was defined as being within 2 meters of another person. Indirect contact was defined as being in the same room but more than 2 meters. In June 2022, participants completed a 2-day individual-level contact diary. We generated age-stratified contact matrices. Contacts were described by contact type, duration and contact relationship. Number and duration of contacts by job were compared by chi-squared test and number of contacts over time by linear regression.

Average contacts of health care worker with all individuals

**Figure:**
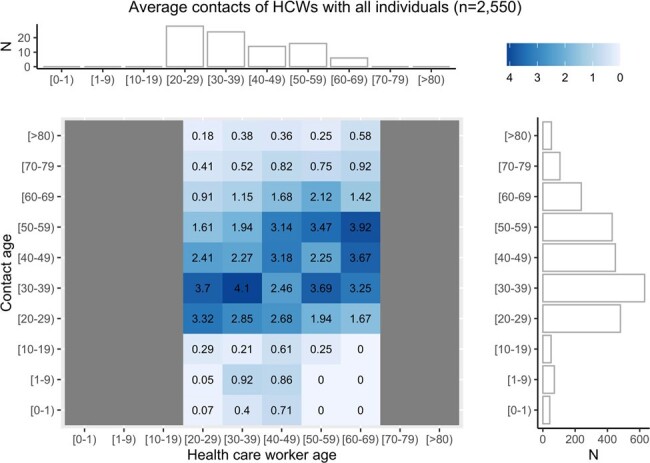

The individual tiles color and numeric fill denote the average number of contacts per HCW of that age range with a contact of that age range. A horizontal marginal histogram shows the age break down of HCW participants and a vertical marginal histogram shows the resulting distribution of contact’s ages.

**Results:**

360 HCWs enrolled, 95 completed 1 or more monthly contact diary and 88 completed the intensive 2-day diary. The total number of contacts was stable over time (β0.14, p 0.20, Fig.1). HCWs reported direct contacts with less than 5 patients, 5-9 patients, 10-19 patients or 20 more patients, 50%, 29%,15% or 6% of the time respectively. HCWs reported direct contact with less than 5 other HCWs, 6-10 HCWs, 11-20 or 21 or more 19%, 39%, 27 % or 15% of the time respectively. In the June 2022, a total of 2,550 contacts were reported, 1,592 (62%) were with HCWs, and 570 (22%) with patients. Social workers, rehab/transport and doctors were more likely to have contact with 20 or more patients vs. other professions. Nurses, imaging technologists and rehab/transport were more likely to spend 20 minutes or more with patients vs. other professions. Contact matrices were not age assortative with HCWs having similar contacts across all working ages (Fig. 2). HCWs’ physical contacts focused on extremes of age (Fig. 3). Contact type varied by job with nearly all groups having more contacts with colleagues than patients (Fig, 4).

Average physical contacts of HCWs per day: The

**Figure:**
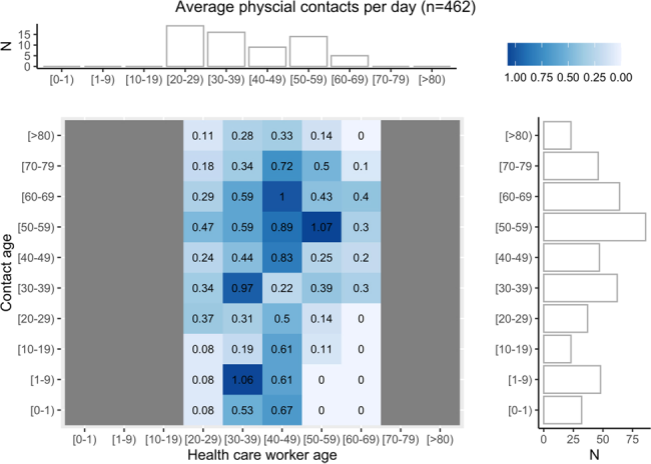

The individual tiles color and numeric fill denote the average number of physical contacts per HCW of that age range with a contact of that age range. A horizontal marginal histogram shows the age break down of HCW participants and a vertical marginal histogram plot the resulting distribution of contact’s ages.

**Conclusion:**

HCW contacts concentrated in their work environment and most contacts were focused in the working-age population. These contacts were stable over time even in the COVID-19 pandemic response. HCWs were 2.8 time more likely to contact HCWs than patients.

Number of contacts of 2 days by participant job and contact individual

**Figure:**
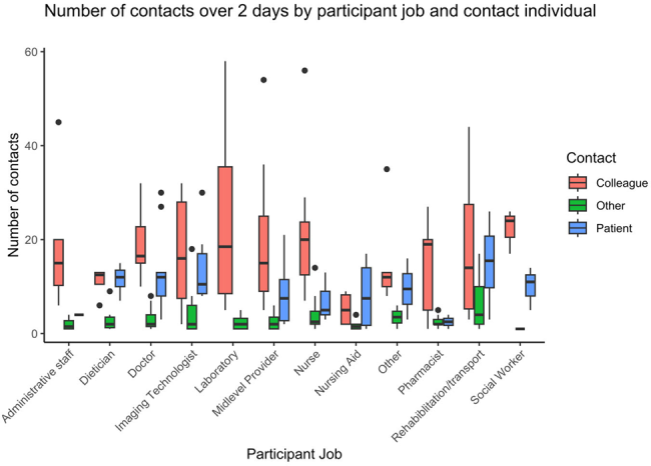

Grouped box plots show the number of contacts over the 2-day intensive diary by HCW job (x-axis) and the HCWs relationship with the contact (e.g. colleague, patient or other).

**Disclosures:**

**Lauren Pischel, MD**, Auxa Health: Advisor/Consultant **Amyn Malik, MBBS, MPH, PhD**, Analysis Group, Inc: Former Employee. We consulted with Pharma and Biotech on different research studies **Ben Lopman, PhD**, Epidemiological Research and Methods, LLC: Advisor/Consultant|Hillevax, Inc: Advisor/Consultant **Albert I. Ko, MD**, Merck: Grant/Research Support|Regeneron: Grant/Research Support **Saad Omer, MBBS MPH PhD**, Meta: Advisor/Consultant|Meta: Grant/Research Support

